# The influence of hepatitis C virus infection on H1 antihistamine treatment in urticaria patients

**DOI:** 10.1186/1471-2334-14-S7-P70

**Published:** 2014-10-15

**Authors:** Lucia Dinu, Corina Daniela Ene (Nicolae), Ilinca Nicolae, Simona Roxana Georgescu

**Affiliations:** 1 Department of Dermatology, MedLife Clinic, Bucharest, Romania; 2 Department of Pharmacology, Carol Davila University of Medicine and Pharmacy, Bucharest, Romania; 3 Department of Research, Clinical Hospital of Infectious and Tropical Diseases “Dr. Victor Babeş”, Bucharest, Romania; 4 Department of Dermatology, Carol Davila University of Medicine and Pharmacy, Bucharest, Romania; 5 Department of Dermatology, Clinical Hospital of Infectious and Tropical Diseases “Dr. Victor Babeş”, Bucharest, Romania

## Background

Considerable evidence indicates that, in addition to anti-allergic effect, several H1-antihistamines also possess anti-inflammatory properties. The anti-inflammatory activity of H1 antihistamine treatment in urticaria patients is based on the capacity of H1-antihistamines to inhibit the release of chemical mediators from mast cells and basophiles, to regulate the chemotaxis of neutrophils and eosinophils, to increase eosinophils apoptosis and to reduce the expression of the adhesion molecules. Viral hepatic infections may affect the efficacy of H1 antihistamines probably interfering with their hepatic metabolism through cytochrome P450 system. We proposed to analyze the effect of hepatitis C virus (HCV) infection on the therapeutic efficacy of H1 antihistamines in urticaria patients.

## Methods

The study included 37 acute and chronic spontaneous urticaria patients divided into two groups (A,B) depending on the associated HCV infection. Group A consisted of 30 urticaria patients without HCV infection and group B included 7 urticaria patients associating HCV infection. The experimental analysis targeted the dynamic of urinary histamine level (spectrofluorimetric method) depending on the Urticaria Activity Score (UAS) and C-reactive protein (CRP) level in patients with urticaria, during the treatment with H1-antihistamines. The clinical and paraclinical evaluations were done at the study entry and at 2 weeks after initiating the H1-antihistamine treatment.

## Results

We obtained much stronger correlations between urinary histamine level and UAS, respectively CRP, for patients in group A (r=0.924, p<0.05, respectively r=0.548, p<0.05 at study entry and r=0.511, p<0.05, respectively r=0.286, p<0.05 after two weeks of H1-anti histamine treatment) comparing to those in group B (r=0.836, p<0.05, respectively r=0.491, p<0.05 at study entry and r=0.484, p<0.05, respectively r=0.265, p<0.05 after two weeks of H1-anti histamine treatment) at both times of the assessment.

## Conclusion

HCV infection reduces the anti-inflammatory effect of H1-antihistamines in urticaria patients.

